# Successful Treatment of Aortic Endocarditis by *Achromobacter xylosoxidans* with Cefiderocol Combination Therapy in a Non-Hodgkin Lymphoma Patient: Case Report and Literature Review

**DOI:** 10.3390/antibiotics11121686

**Published:** 2022-11-23

**Authors:** Gianfranco La Bella, Francesco Salvato, Graziano Antonio Minafra, Irene Francesca Bottalico, Tiziana Rollo, Lucia Barbera, Samantha De Tullio, Gaetano Corso, Sergio Lo Caputo, Rosella De Nittis, Fabio Arena

**Affiliations:** 1 Department of Clinical and Experimental Medicine, University of Foggia, 71122 Foggia, Italy; 2 Istituto Zooprofilattico Sperimentale della Puglia e della Basilicata, 71121 Foggia, Italy; 3 AOU Policlinico “Riuniti”, 71121 Foggia, Italy; 4 Department of Medical and Surgical Science, University of Foggia, 71121 Foggia, Italy

**Keywords:** bloodstream infections, Gram-negative, multi-drug resistance, siderophore, cephalosporins

## Abstract

*Achromobacter xylosoxidans* is a Gram-negative aerobic opportunistic bacterium, belonging to the order of *Burkholderiales*, that can cause infections of virtually all body districts in patients with underlying diseases. However, *A. xylosoxidans* has rarely been associated with infective endocarditis. The treatment of *A. xylosoxidans* infections is complicated by both intrinsic and acquired resistance. Here we report on a case of aortic endocarditis by *A. xylosoxidans* in a Non-Hodgkin lymphoma patient treated with a combination of cefiderocol and other antibiotics, and summarize the available literature.

## 1. Introduction

*Achromobacter xylosoxidans* is a Gram-negative, aerobic, oxidase and catalase positive bacterium, belonging to the order of *Burkholderiales*. It is an environmental species, found in humid environments and can also be isolated in the hospital setting and, in some circumstances, poses a health risk, being an agent of infections in patients with co-morbidities [1]. *A. xylosoxidans* can cause infections in immunocompromised patients, those with respiratory diseases (i.e., patients with cystic fibrosis) and with invasive devices. Rarely, endocarditis related to *A. xylosoxidans*, correlated with high mortality, has been reported [2].

*A. xylosoxidans* is constitutively resistant to many antibiotics, including cephalosporins, ertapenem and aztreonam (due to the presence of an OXA-type, an AmpC type intrinsic β-lactamase and multi-drug efflux systems) and can acquire transferable resistance genes; therefore, infections caused by this germ can be difficult to treat due to limited therapeutic options [1,3,4]. Moreover, biofilm formation, which appears to be an intrinsic ability of most of the strains, contributes to antimicrobial resistance and virulence [5]. Therefore, it is important to evaluate the activity of new antibiotic molecules against bacteria belonging to this species.

Cefiderocol is a siderophore cephalosporin able to bind to extracellular free iron; this allows the active transport of the drug in the periplasmic space of Gram-negative bacteria. Once it reaches the periplasmic space and the PBPs, cefiderocol inhibits the synthesis of peptidoglycan and the bacterial cell wall assembly, resulting in cell lysis and death. Cefiderocol is licensed for rescue therapy of infections due to aerobic Gram-negative organisms in adults with limited treatment options [6].

In this report we present a case of *A. xylosoxidans* endocarditis of a native aortic valve in an immunocompromised patient with Non-Hodgkin lymphoma, treated with cefiderocol with bacteriological and clinical success.

## 2. Case Report

In February 2022, a 57-year-old male patient with an history of high-grade B-cell Non-Hodgkin lymphoma (Diffuse Large B Cell Lymphoma, stage IVB, treated with EPOCH regimen, with a partial response; diagnosis November 2021), aneurysm of the left basilar artery and recent bilateral peritonsillar abscess presented a sepsis caused by *A. xylosoxidans* during hospital admission in the haematology ward. The episode was related to an infection of an endovascular device (central venous catheter). The device was promptly removed and the patient was treated with piperacillin/tazobactam (4.5 gr. IV q6hr for 10 days) with clinical improvement, followed by hospital discharge. After two months of clinical stability, in April 2022 the patient referred to the Emergency Department for persistent hyperpyrexia (10 days), despite therapy with ceftriaxone. The nasopharyngeal swab for research of SARS-CoV-2 was negative. Blood tests showed increased C-reactive protein (CRP) and erythrocyte sedimentation rate (ESR) with negative procalcitonin. At chest X-ray, multiple inflammatory foci were present and the echocardiogram showed a vegetation of the aortic valve. Blood culture was positive for *A. xylosoxidans* with a multi-drug resistant profile (Table 1).

After initial empiric antibiotic therapy with piperacillin/tazobactam (4.5 gr. IV q6hr) and teicoplanin (12 mg/kg IV q12hr), followed by imipenem (500 mg IV q6hr ), the patient was transferred to the infectious diseases ward for positivity to SARS-CoV-2. The therapy was modified to meropenem (2 gr. IV q8hr), fosfomycin (4 gr. IV q6hr), trimethoprim/sulfamethoxazole (160/800 mg q12hr orally) and daptomycin (8 mg/kg IV q24hr). Anti-MRSA coverage was maintained considering the site of infection and the features of the patient.

**Figure 1:**
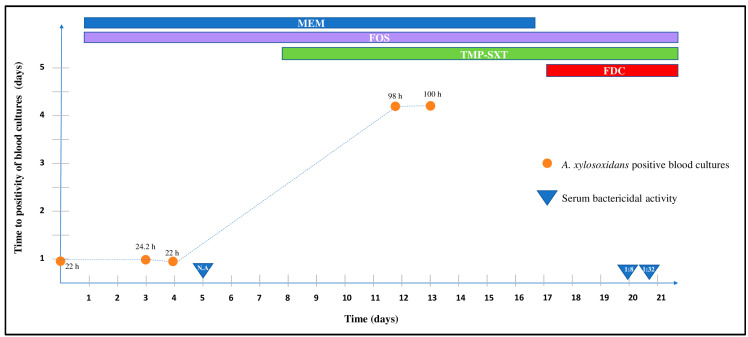
Summary of the therapeutic regimen and bacteriological tests carried out during the infectious diseases ward stay. N.A.: no activity; MEM: meropenem; FOS: Fosfomycin; TMP-SXT: trimethoprim-sulfamethoxazole; FDC: cefiderocol.

Despite this therapy, which induced a progressive increase in the time to a positivity of blood cultures (from 22 h to >4 days) (Figure 1), the patient continued to present fever with shaking chills, chest pain, weight loss, breathlessness, and rapid heart rate.

For this reason, after 17 days, the antibiotic therapy was modified, interrupting meropenem and introducing cefiderocol. Concurrently, due to the onset of acute respiratory distress syndrome (ARDS), probably SARS-CoV-2 related, remdesivir and corticosteroids were introduced.

After modification of the antibiotic therapy with the addition of cefiderocol (2 gr. IV q8hr over 3 hr), the patient showed a slow clinical improvement. Blood cultures, after the introduction of cefiderocol, were repeatedly negative. Since the introduction of cefiderocol, the bactericidal activity of the serum increased from no activity (pre-cefiderocol) to a dilution of 1:8 (for trough sampling) and 1:32 (for peak sampling). The patient had a favourable response to the antibiotic course with apyrexia, respiratory improvement, progressive pain decrease and biochemical inflammation markers showing normalization. Improvements could be partially secondary to remdesivir and corticosteroids introduction for SARS-CoV-2 infection management.

## 3. Discussion and Conclusions

*Achromobacter xylosoxidans* is a Gram-negative aerobic bacterium that was first described in 1971 by Yabuuchi and Oyama in patients with chronic, purulent otitis media, and has been associated with nosocomial infections in immunocompromised patients [7]. *A. xylosoxidans* has previously been described as a cause of bacteremia, meningitis, otitis media, urinary tract infections, pneumonia and, rarely, prosthetic infections [8,9]. Cardiac involvement with endocarditis has been only occasionally reported, especially in native valves [10]. Although rare, endocarditis due to *A. xylosoxidans* is often fatal, mostly due to the limited therapeutic options available.

In this paper we describe a case of native aortic endocarditis caused by *A. xylosoxidans* in an immunocompromised patient successfully treated with cefiderocol based therapy. To our knowledge, only 16 other cases of *A. xylosoxidans* endocarditis involving the aortic valve have been described in the literature so far [10,11,12,13,14,15,16,17,18,19,20,21,22,23,24,25] (Table 2), and none treated with cefiderocol.

The mean age of infected patients was 54 years (range, 19–86 years) and in all cases significant comorbidities were reported. The overall mortality rate for *A. xylosoxidans* endocarditis, involving the aortic valve, was 56.2%. In one case the outcome was not reported. The mortality rate was different among patients receiving antibiotics as well as surgery (16.6%; 1/6), and patients treated with antibiotics alone (80%; 8/10). An 86-year-old woman and the patient of our case (57-year-old man) were the only two patients who survived with medical treatment alone (Table 2). Although these data seem to support the importance of valve replacement, surgery is not always possible (e.g., operative risk and comorbidities). These data confirm the challenge posed by *A. xylosoxidans* and suggest that an appropriate and prompt antibiotic treatment, and a combination of antibiotics, may be important determinants of survival.

Recent articles have reported the treatment with cefiderocol of intravascular devices infections or endocarditis caused by other extensively drug-resistant (XDR) pathogens. A case of XDR *Acinetobacter baumannii* causing pneumonia and bloodstream infection in a patient on extracorporeal membrane oxygenation was reported [26]. Edgeworth and colleagues [27] reported a case of a 78-year-old woman with hospital-acquired native aortic valve endocarditis due to XDR *Pseudomonas aeruginosa*, who was successfully treated with an antibacterial combination therapy with cefiderocol, colistin, and meropenem. Although the patient underwent aortic valve surgery after six doses of cefiderocol, the valve culture was negative.

A possible advantage of cefiderocol is activity against biofilm-forming Gram-negative pathogens, as shown by a recent report suggesting that cefiderocol may reduce formed biofilm by Gram-negative bacteria [28].

In our case, the activity of cefiderocol has been shown also by a serum bactericidal test (SBT). The SBT is a semiquantitative assay that measures the highest dilution of patient serum sampled during antibiotic treatment that kills >99.9% of a bacterial inoculum within 24 h [29]. Although it has methodological limitations, SBT is the only test that combines microbiological, pharmacokinetic and pharmacodynamic parameters and could play a renewed role in the monitoring of antibiotic therapy for multidrug-resistant Gram-negative infections [30]. SBT can be an important support while introducing new drugs, such as cefiderocol, in the clinical practice.

To the best of our knowledge, this case represents the first report of infective endocarditis caused by *A. xylosoxidans*, involving a native aortic valve, treated successfully with cefiderocol combination therapy, without the necessity of valve replacement surgery. Given the significant associated morbidity and mortality, along with a high degree of intrinsic antibiotic resistance, *Achromobacter* species infective endocarditis remains a clinical treatment challenge. Cefiderocol can be considered an additional, promising option for salvage therapy of *Achromobacter* endocarditis, even when surgery is not possible.

## 4. Materials and Methods

The bacterial strain was isolated from blood samples using the BD BACTEC™ FX system (Becton, Dickinson and Company, Franklin Lakes, NJ, USA). The strain grew in the aerobic blood culture bottle and became positive after 22 h of incubation. The isolate was subjected to identification at the species level by MALDI-TOF technology (Bruker, Billerica, MA, USA).

Antimicrobial susceptibility testing was performed by the reference microdilution method according to the CLSI document M07Ed11 [31] with the following antibiotics: amikacin, cefepime, ceftazidime, ceftazidime/avibactam, ceftolozane/tazobactam, ciprofloxacin, colistin, gentamicin, levofloxacin, meropenem, minocycline, piperacillin/tazobactam, tigecycline, trimethoprim/sulfamethoxazole. The susceptibility testing to fosfomycin was performed by the agar-dilution method. Cefiderocol was tested by both microdilution and disc-diffusion methods (Liofilchem, Roseto degli Abruzzi, Italy), which resulted in an MIC of 1 mg/L and an inhibition zone of 35 mm, respectively. The minimal inhibitory concentrations (MICs) are presented in Table 1. Interpretation was according to the most recent EUCAST breakpoints v_12.0 [32]. In the absence of breakpoints, the PK/PD criteria were applied. For the interpretation of the cefiderocol test, the cut-off ≥ 17 mm corresponding to the PK/PD breakpoint of S ≤ 2 mg/L was used.

The serum bactericidal activity test was carried out in accordance with the CLSI procedure [33]. Serum samples were drawn: (i) before the start of cefiderocol treatment; (ii) during cefiderocol treatment, at the steady state, just before one administration (trough level); (iii) one hour after the end of the administration (peak sampling).

## Figures and Tables

**Table 1:** Antimicrobial susceptibility testing results for the isolate included in the study.

Antibiotic	MIC Value (mg/L)	Interpretation EUCAST (S/I/R)
Amikacin	8	R *
Cefepime	>16	R *
Cefiderocol	1	S *
Ceftazidime	8	I *
Ceftazidime/avibactam	4/4	S *
Ceftolozane/tazobactam	8/4	R *
Ciprofloxacin	0.5	I *
Colistin	1	IE
Fosfomycin	128	IE
Gentamicin	>8	R *
Levofloxacin	≤4	R *
Meropenem	2	I
Minocycline	≤4	IE
Piperacillin/tazobactam	≤2/4	S
Tigecycline	≤0.25	S *
Trimethoprim/Sulfamethoxazole	≤1/19	S

S: susceptible, standard dosing regimen; I: susceptible, increased exposure; R: resistant; * PK/PD criteria applied; IE: insufficient evidence.

**Table 2:** Summary of previously reported *Achromobacter* spp. endocarditis involving aortic valve.

Author (Ref)	Year	Age/Sex	Risk Factor	Comorbidity	Affected Valve	Valve Prosthesis	Antibiotics Prescribed	Surgery	Outcome
Cole et al. [11]	1952	54 yrs/F	VD	DM, malaria	Aortic	No	Penicillin, chloramphenicol, streptomycin, sulphadiazine	No	Died
Lofgren et al. [12]	1981	77 yrs/F	PrV	Rheumatic heart disease PrV	Mitral and aortic	Yes for PrV only	Tobramycin, carbapenicillin trimethoprim/sulfamethoxazole, moxalactam	No	Died
Davis et al. [13]	1982	30 yrs/M	MS	MS, HF	Aortic	NR	NR	No	Died
Olson et al. [14]	1982	35 yrs/M	Dissection of the aortic root; AVR + graft	Prosthetic valve; Chronic IV access	Aortic	Yes	Carbenicillin, trimethoprim/sulfamethoxazole, rifampicin, moxalactam, azlocillin	No	Died
Valenstein et al. [15]	1983	62 yrs/M	VD		Aortic	No	Cefazolin, gentamicin	No	Died
McKinley et al. [16]	1990	28 yrs/M	AVR + graft		Aortic	Yes	Cefuroxime, gentamicin	Yes	Survived
Sasaki et al. [17]	1993	39 yrs/M	VSD (post operation)	Patch closure of VSD Dental treatment	Aortic	Yes	Piperacillin, amikacin, cefotetan, sulbactam/cefoperazone, fosfomycin, enoxacin, cefpiramide, ceftazidime, minocycline	Yes	Survived
Nanuashvili et al. [18]	2007	46 yrs/M	NR	DM, IS, emphysema	Mitral Aortic	NR	Ampicillin, tazobactam, trimethoprim/sulfamethoxazole	Yes	Survived
Van Hal et al. [19]	2008	37 yrs/M	PrV, IHD	Intravenous drug user	Aortic	Yes	Carbapenem	Yes	Survived
Ahmed et al. [20]	2009	69 yrs/M	PrV	DM, CABG, H	Mitral and aortic	Yes for aortic only	Ertapenem, tigecycline, trimethoprim/sulfamethoxazole	Yes	Died
Storey et al. [21]	2010	79 yrs/F	None	AF, TIA, H	Mitral and aortic	No	Meropenem	No	Died
Tokuyasu et al. [22]	2011	86 yrs/F	PrV	NR	Aortic	Yes	Carbapenem	No	Died
Rodrigues et al. [10]	2017	86 yrs/F	NR	PF, IHD, CKD	Aortic	No	Piperacillin/tazobactam+ trimethoprim/sulfamethoxazole	No	Survived
Kumar et al. [23]	2017	54 yrs/M	None	CKD, H	Mitral and aortic	No	Piperacillin/tazobactam	No	NR
Xia et al. [24]	2018	66 yrs/F	None	H, DM, ESRD	Mitral and aortic		Meropenem, vancomycin, levofloxacin, trimethoprim/sulfamethoxazole	No	Died
de Castro et al. [25]	2020	19 yrs/M	CS, aortic bicuspid	None	Aortic	No	Meropenem	Yes	Survived
This case	2022	57 yrs/M		Non-Hodgkin lymphoma	Aortic	No	Meropenem, fosfomycin, daptomycin, trimethoprim-sulfamethoxazole, cefiderocol	No	Survived

AF: atrial fibrillation; AVR: aortic valve replacement; CABG: coronary artery bypass grafting; CKD: chronic kidney disease; CS: cardiac surgery; DM: diabetes mellitus; ESRD: end-stage renal disease; H: hypertension; HF: heart failure; IHD: ischemic heart disease; IS: ischemic stroke; MS: mitral stenosis; NR: not reported; PF: Pulmonary fibrosis; PrV: prosthetic valve; TIA: transient ischemic attack; VD: valve disease; VSD: ventricular septal defect.

## Data Availability

Not applicable.
